# Innovative Strategies for Remotely Sampling Hard-to-Reach Populations: Assessing Phone Versus Internet Respondent-Driven Sampling Approaches Among Venezuelan Refugees and Migrants in Colombia

**DOI:** 10.1093/aje/kwad116

**Published:** 2023-05-16

**Authors:** Phuong N Pham, Lisa G Johnston, Katrina Keegan, Carol Wei, Patrick Vinck

**Keywords:** migrants, refugees, remote surveys, respondent-driven sampling, Venezuela

## Abstract

It is challenging to quantitatively measure the health vulnerability and risk factors of refugees and migrants residing outside of formal settlement settings. For hard-to-reach populations without available sampling frames, researchers have increasingly turned to novel sampling and statistical methods, like respondent-driven sampling (RDS). “Standard” RDS is typically conducted face-to-face at fixed sites. However, during the coronavirus disease 2019 (COVID-19) pandemic, face-to-face survey methods and recruitment approaches posed high potential risk of virus transmission and infection, making remote RDS approaches optimal. In this paper, we explore the feasibility of implementing telephone and Internet RDS strategies to assess challenges faced by Venezuelan refugees and migrants in the city of Bogotá, Colombia’s capital, and the department of Norte de Santander, the main Venezuelan-Colombian border crossing site. We describe RDS assumptions, survey design, formative research, and the implementation of both strategies and present diagnostics for determining whether assumptions are met. Phone-based recruitment strategies in both locations and the Internet strategy in Bogotá achieved their calculated sample size; however, the Internet strategy in Norte de Santander did not. Most RDS assumptions were sufficiently met at sites where sample sizes were reached. These surveys provide valuable lessons for implementing innovative remote strategies with which to study hard-to-reach populations such as refugees and migrants.

## Abbreviations

COP: Colombian pesos
COVID-19: coronavirus disease 2019
RDS: respondent-driven sampling
UNHCR: United Nations High Commissioner for Refugees

Refugee and migrant populations living in informal settlements are difficult to sample with probability-based methods, because there is no exhaustive listing of group members for random sampling and because they are difficult to identify or recruit using population-based sampling frames (1). Estimating the health vulnerabilities of these populations is often challenging because they may have irregular (undocumented) legal status or may be highly stigmatized or victimized (e.g., targets of xenophobia, victims of labor exploitation or abuse, survivors of sexual violence, etc.). Confronted with similar challenges, researchers have deployed new sampling and statistical techniques such as respondent-driven sampling (RDS) among people who use drugs, sex workers, or other groups at high risk for human immunodeficiency virus transmission (1, 2). More recently, RDS has been used successfully among refugee and migrant populations (3). RDS is a link-tracing sampling method which initiates sampling with an eligible participant, known as a “seed,” who has a large and diverse social network. After completing the survey, the seed receives recruitment coupons which are used to recruit other eligible peers from their social network to participate in the survey. This peer-to-peer recruitment process continues for multiple waves of recruitment until the sample size has been reached. RDS requires adherence to multiple network sampling and statistical theories to mitigate the biases commonly found in peer-to-peer recruitment (4). Such requirements include having a recruitment quota (i.e., each participant can only recruit a minimum number of people, usually 3, and the maximum number of recruits must be equal for all), maintaining long recruitment chains to help minimize bias (i.e., the number of waves reaches a value large enough to eliminate bias from the initial seed selection), and ongoing improvement in estimators that address issues of differential recruitment, without replacement sampling and the treatment of reported network sizes (5, 6).

Since the debut of RDS in 1997, the method has been widely applied (2, 7) in person, whereby potential research participants (recruits) present a physical coupon and are screened, interviewed, and provided with incentives and recruitment coupons in person at a fixed site. With the onset of the coronavirus disease 2019 (COVID-19) pandemic, face-to-face survey methods and recruitment posed high potential risk of virus transmission and infection due to close physical contact. Thus, remote approaches such as telephone or Internet recruitment presented alternative approaches for sampling hard-to-reach populations. Remote RDS surveys face several challenges, including sampling across an incomplete social network component (i.e., the social network is made up of subgroups that are unconnected within the larger network being sampled), slow recruitment, inability to reach the calculated sample size, duplicate enrollment or enrollment of ineligible persons, the need to develop new software protocols and programming to monitor recruitment and the connections between recruits and recruiters, and lack of a recruitment quota (8–12). Phone and Internet RDS approaches have been used with varying degrees of success to sample the general population and men who have sex with men (8–12). To the best of our knowledge, the current study represents the first successful use of remote RDS among refugee and migrant populations.

In this paper, we evaluate and compare telephone and Internet RDS strategies among Venezuelan refugees and migrants in the city of Bogotá, the capital of Colombia, and the department of Norte de Santander (Colombia has 32 departments, which are administratively similar to states in the United States), where the majority of Venezuelan refugees and migrants physically cross into Colombia. We present methods for optimizing remote RDS, including assessing social networks through formative research, using a novel method of “directed seed” recruitment to ensure sociometric depth and minimize bottlenecks (i.e., network subgroups that may not be well connected in the larger network), and using diagnostics of estimates to assess homophily and convergence (13). We present methods for mitigating biases inherent in network sampling, including operationalizing key questions for determining the network structure of a refugee and migrant population, seed selection and direction, and appropriate analysis. Finally, we present results of comparisons of the demographic characteristics of refugees and migrants by site, data collection method, and recruitment status (recruiters vs. nonrecruiters).

## METHODS

Bogotá and Norte de Santander were selected primarily because of their large Venezuelan refugee and migrant populations, which combined host an estimated 30% of Venezuelan refugees and migrants in Colombia. The majority of Venezuelan refugees and migrants own a smartphone (70%) and have access to the Internet (79%)—a strong indication of the potential success of both phone and Internet RDS (14). In Colombia, Internet access in Bogotá (81%) is greater than in Norte de Santander (74%) (15).

### Formative research

Formative research to assess the feasibility of RDS and the network structure of the targeted population is crucial to ensure recruitment success and data quality (16, 17). It is essential to assess the network structure of a hard-to-reach population to determine whether sampling can be sustained through peer-to-peer recruitment. Formative research measures whether a population composes one complete network or is made up of several independent subgroups (i.e., different languages), engages in social activities through work and/or socializing, and, if invited by a peer to take part in a survey, would participate (17). In this study, formative research involved consultations to gain knowledge of the population’s social networks, identify potential bottlenecks, and interviews with Venezuelan refugees and migrants to gauge the size of their social networks, ability to recruit within the eligibility criteria and across subgroups and targeted vulnerability profiles, and willingness to participate in surveys. In addition, the team reviewed available data regarding the target population’s location, access to phones and/or Internet, and survey response rates. Although there was some evidence that the population might participate in either a phone or Internet RDS, there was no clear evidence that either method would be successful.

### Quantitative research (RDS)

Eligible persons were those who were 18 years of age or older, had been born in Venezuela, had lived or worked in either Bogotá City or Norte de Santander Department for 1 month or more, and had arrived in Colombia after 2014. A sample size of 600 for each region in the country was calculated using a design effect of 2, 80% statistical power with a 2-sided test, and α = 0.05 to detect a decrease of 10%, where 30% was assumed to be the percentage of persons estimated to have an irregular legal status at time 1 (this survey) and 20% was assumed to be the percentage at time 2 (the next round of this survey). All surveys (phone and Internet in Bogotá and Norte de Santander; *n* = 4 with a target sample size of 300 each) began with 3 seeds. Seed participants were selected with the help of organizations in Colombia who work with Venezuelan refugees and migrants. Seed participants were directed to recruit across potential bottlenecks (18, 19) identified through formative research. Web Table 1 describes the phone and Internet RDS implementation steps and lessons learned.

Local organizations identified and facilitated contact with potential seeds. Potential seeds were interviewed about their peer network using a “diversity recruitment grid” listing characteristics such as age, education, civil status, contact with assistance organizations, neighborhood of residence, and vulnerability profiles (Web Table 2). Final seeds were selected on the basis of their ability to recruit across diverse eligible members of their network. Directing seeds to recruit across bottlenecks ensures that bias stays with the seeds (versus adjusting once sampling is under way) and that a diverse mix of people enroll in the survey early on (19).

Phone RDS was conducted by trained interviewers who contacted respondents by phone and conducted the survey verbally. After the survey, interviewers explained the recruitment process and asked whether participants could recommend 3 peers who met the eligibility criteria. If participants agreed, they were asked to share the phone numbers of their 3 recruits. Recruits were then contacted by phone.

For Internet RDS, respondents received an invitation and a unique survey link to complete an Internet survey. The form included instructions, definitions, and appropriate skip logic. Upon completing the survey, participants read recruitment instructions and shared the mobile phone contact information of up to 3 eligible peers. Recruits were then sent a unique invitation and survey link so they could participate as well.

Incentives were provided to participants for completing the survey and for successful recruitment. Participants received mobile phone credits worth no more than the cost of lunch, sent directly and remotely to the participant within 30 hours of having successfully completed the survey. A primary incentive of 12,000 Colombian pesos (COP; approximately US$3.00) was provided for completion of the survey, and smaller secondary incentives of 3,000 COP (approximately US$0.75) were given for each eligible recruit (for up to 3 recruits per person) who also completed the survey.

The survey questionnaire was written in English and translated into Spanish. After translation, the survey was pretested in 2 stages. First, the United Nations High Commissioner for Refugees (UNHCR) expert team in Colombia reviewed the surveys and validated the content, clarity, and appropriateness of question wording and translation. Second, the survey was built in KoboToolbox (Kobo Inc., Cambridge, Massachusetts), and the research team pretested it online and by phone with 1 seed, out to 3 waves, to test feasibility. Then the surveys were scaled up by adding 2 additional seeds to each study to reach a minimum of 3 seeds per location and approach (as per the study protocol of a minimum of 1 seed per 100 sample size). During data collection, in an effort to increase recruitment, numbers of seeds were increased to 6 for both Bogotá and Norte de Santander phone RDS and to 5 for Bogotá Internet RDS and 10 for Norte de Santander Internet RDS. For phone RDS, interviewers verbally administered the survey over the phone in Spanish and recorded responses in the online KoboToolbox form on computers. Internet RDS was self-administered by participants by opening the survey link and recording their answers directly in the online KoboToolbox form on their personal mobile phones or computers. Phone and Internet RDS used the same questionnaire, though instructions and hints were tailored toward each survey strategy. The survey contained questions related to participants’ personal situation and experiences, including sociodemographic characteristics, migration patterns, legal status, living/housing situation, exposure to violence, health conditions and access to health care, and disability; their access to and perceptions about assistance and information; and their knowledge, perceptions, and behaviors surrounding COVID-19.

This study protocol was reviewed by the Partners Human Research Committee and the Partners’ Research Information Security Office (Mass General Brigham, Boston, Massachusetts) and was ultimately deemed to be not human subjects research but an internal UNHCR evaluation. All participants gave informed consent and were provided links to a website with organization information, contact information, and a list of UNHCR resources.

### Data management and analysis

A cloud collaboration service, Airtable (Airtable, San Francisco, California), was used to store and track participant identification numbers, recruit identification numbers, participant status, contact information, and incentive information. Five percent of phone RDS data was subject to fidelity monitoring, comparing the survey output with interview recordings. Data were entered, merged, analyzed, and stored on the research team computers. Data management and security procedures are summarized in Web Table 3.

Univariate analysis with population estimates and 95% confidence intervals were computed using Giles successive sampling estimator in RDS Analyst (6). Biases associated with peer-to-peer recruitment are mitigated by weighting the data by differential network sizes during statistical analysis (4). These weights are developed by asking each participant the size of their social network. Frequencies, weighted percentages, and 95% confidence intervals were calculated using exported network weights and survey data analysis in STATA, version 13 (StataCorp LLC, College Station, Texas). Second-order Rao-Scott corrections (20) were used to account for survey design in tests of independence comparing demographic characteristics of recruiters versus nonrecruiters.

Several diagnostic procedures (13) for testing RDS assumptions and for assessing the level of bias in the final estimates are available (21, 22). The assumption that the final sample is not dependent on the seeds was assessed through convergence (13). Convergence is defined as the point in recruitment whereby the final adjusted estimate no longer changes and remains stable over several additional waves of recruitment. When estimates shown in convergence plots do not level off as the sample size increases, the current estimate is likely to have been biased by the choice of seeds. The assumption that the sample is composed of 1 complete network component was tested using recruitment graphics and bottleneck plots. If subgroups differ with respect to the population characteristics of interest, then population estimates may also be biased. Independence among subgroups was tested using recruitment and population homophily. Recruitment homophily measures the tendency of individuals within a group to be connected to individuals of that same group during the recruitment process, and population homophily measures the tendency to recruit people with similar traits within the population. Homophily is defined here as the ratio of the number of within-group ties to the number that would be expected if there were no homophily (23). For this study measurement, a value of 2 indicates twice as many ties as expected, and a value of 1 indicates no homophily. See Web Table 4 for a description of these assumptions, the diagnostic used, its definition and importance, and problems that arise if these assumptions are not met

**Table 1:** Distribution of Respondent Types (Number of Persons) by Respondent-Driven Sampling Strategy in a Study Carried Out Among Venezuelan Refugees and Migrants, Bogotá and Norte de Santander, Colombia, 2020

	**RDS Strategy**			
	**Phone RDS**		**Internet RDS**	
**Respondent Type**	**Bogotá** **(*n* = 550)**	**Norte de Santander** **(*n* = 531)**	**Bogotá** **(*n* = 578)**	**Norte de Santander** **(*n* = 91)**
Participants	305	300	302	35
Recruiter^a^	230	223	235	28
Nonrecruiter^b^	75	77	67	7
Nonparticipants	245	231	276	56
Never reached	112	84	191^c^	44^d^
Ineligible	102	108	82	12
Refused participation	27	34	0	0
Did not consent	1	2	3	0
Did not finish	3	3	0	0

Abbreviation: RDS, respondent-driven sampling.

^a^ Recruiters were defined as those who decided to recruit additional participants and who provided the phone numbers of recruits during the survey (regardless of whether or not those recruits went on to participate).

^b^ Nonrecruiters were those who decided not to recruit additional participants.

^c^ A total of 171 persons were never reached after 3 attempts; 20 were never reached because there was no WhatsApp corresponding to the phone number provided.

^d^ A total of 37 persons were never reached after 3 attempts; 7 were never reached because there was no WhatsApp corresponding to the phone number provided.

## RESULTS

**Figure 1:**
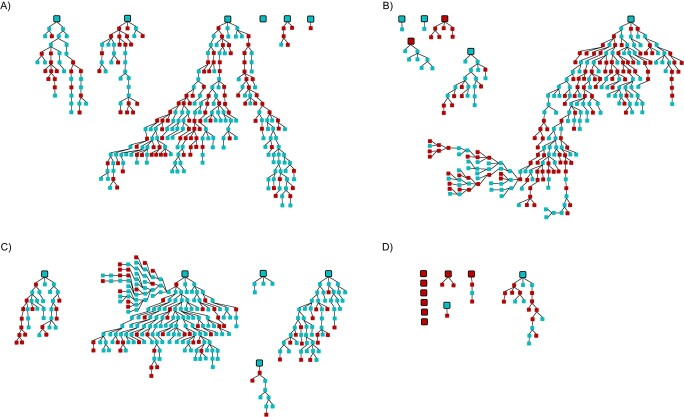
Recruitment chains for each seed participant (blue, women; red, men) in a study of respondent-driven sampling (RDS) strategies among Venezuelan refugees and migrants, Bogotá and Norte de Santander, Colombia, 2020. Large squares at the beginning of a chain represent seeds, and small squares represent recruits. A) Bogotá telephone RDS; B) Norte de Santander telephone RDS; C) Bogotá Internet RDS; D) Norte de Santander Internet RDS.

Phone RDS was conducted between August and September 2020, and Internet RDS was conducted between October and November 2020. The final sample sizes for the phone surveys were 305 in Bogotá and 300 in Norte de Santander, and the final sample sizes for the Internet surveys were 302 in Bogotá and 35 in Norte de Santander (Table 1). Response rates ((total study community − never reached)/total study community) for the phone surveys were 80% in Bogotá and 84% in Norte de Santander, and the response rate for the Bogotá Internet survey was 67%. The completion rates (total participants/total study community) for the phone surveys were 55% in Bogotá and 56% in Norte de Santander, and the completion rate for the Bogotá Internet survey was 52%. Recruitment rates (total recruiters/total participants) in the phone surveys were 75% in Bogotá and 74% in Norte de Santander, and the recruitment rate for the Bogotá Internet survey was 78%. The maximum number of waves reached for the phone surveys was 22 in Bogotá and 28 in Norte de Santander, and the maximum number of waves for the Internet surveys was 12 in the Bogotá and 8 in Norte de Santander. Figure 1 shows the recruitment graphics initiated by each seed according to sex, location, and RDS approach, which is helpful for identifying clustering among traits (e.g., males only recruiting males). We found males and females mixed throughout the chains and no indication of clustering or bottlenecking.

To identify barriers to participation for the Internet survey in Norte de Santander, nonrespondents who were sent the invitation via WhatsApp (Meta Platforms, Inc., Menlo Park, California) were contacted by phone to complete a 5-minute or less verbal survey. Five call attempts were made to reach nonrespondents. Of the 37 nonrespondents, 26 (70%) were contacted and asked whether they had received the invitation and, if so, to provide the reason(s) they did not participate. Of the 26 nonrespondents contacted, 7 (27%) reported that they had never received the link. Of the 19 who had received the link, the 2 most common obstacles were not having Internet access with which to open and/or send the survey (58%) and not understanding the survey (37%).

For the phone recruitment method at both study sites, there were no statistically significant differences in demographic and vulnerable characteristics among recruiters and nonrecruiters except for age in Norte de Santander, where nonrecruiters tended to be older than recruiters. Web Table 5 gives the demographic characteristics of recruiters and nonrecruiters in Bogotá to provide data on potential bias resulting from loss of potential participants from nonrecruiters’ networks. With respect to Internet RDS in Bogotá, recruiters were more likely than nonrecruiters to be female (82.3% vs. 55.2%; 2-sided *P* = 0.002), single (82.0% vs. 70.1%; 2-sided *P* = 0.049), and undocumented (59.9% vs. 30.3%; 2-sided *P* = 0.008). There were no significant differences between recruiters and nonrecruiters for phone RDS. Overall, participants in phone RDS were more likely than participants in Internet RDS to be male (41.6% vs. 25.5%; *P* = 0.008) and living with a partner (60.4% vs. 42.2%; *P* = 0.005).

Table 2 presents the results of homophily tests for phone RDS versus Internet RDS in Bogotá according to 5 variables. These variables were selected because our primary interest in the study was to describe the demographic profiles of vulnerable refugees and migrants and demonstrate diversity in diagnostic findings. Overall, homophily hovered around 1, indicating no preferential recruitment of subjects with certain traits, conditional on the existing social ties of those recruited. The Bogotá Internet survey demonstrated some population homophily for persons living with a partner and for those who were married, indicating some preferential recruitment within those traits. It may be that people living together preferentially selected their partner to participate in the survey. Single people in the Bogotá Internet survey had a population homophily of 1.39, indicating that single people had 39% more connections to other single people than to partnered people. Some population homophily was also evident in the Bogotá phone survey for legal status, indicating some preferential recruitment within each legal status category.

**Table 2:** Results of a Homophily Test for the Demographic Characteristics of Telephone RDS Participants Compared With Internet RDS Participants, Bogotá, Colombia, 2020

**Variable and** **RDS Strategy**	**No. of** **Persons**	**Sample Size** **(No.)**	**Point Estimate, %**	**95% CI**	**Estimated Design Effect (SE)**	**Recruitment Homophily**	**Population Homophily**
Female sex							
Phone	302	175	62.2	52.5, 71.8	3.1 (4.95)	1.19	1.20
Internet	298	220	72.2	63.8, 80.5	2.7 (4.27)	1.08	1.05
Living with a partner (yes)							
Phone	302	180	58.1	49.5, 66.6	2.4 (4.35)	1.07	1.06
Internet	297	133	40.9	32.5, 49.3	2.3 (4.29)	1.27	1.35
Civil status							
Phone	297						
Divorced, separated, or widowed		12	3.5	0, 7.0	3.0 (1.82)	1.08	1.02
Married or in a civil union		91	28.8	21.1, 36.6	2.2 (3.94)	1.08	1.06
Single		194	67.7	60.1, 75.2	2.0 (3.84)	1.08	1.09
Internet	293						
Divorced, separated, or widowed		6	1.0	0, 2.3	1.5 (0.70)	1.11	0.83
Married or in a civil union		59	18.4	11.6, 25.3	2.4 (3.49)	1.11	1.27
Single		228	80.6	73.7, 87.5	2.3 (3.54)	1.11	1.39
Low educational level (secondary school or less)							
Phone	302	65	28.0	20.9, 35.1	2.0 (3.63)	1.04	1.03
Internet	299	89	37.8	31.4, 44.1	1.3 (3.25)	1.10	1.16
Legal status							
Phone	295						
Irregular^a^		131	50.9	41.8, 59.9	2.5 (4.61)	1.22	1.29
Regular/refugee/seeking asylum^b^		164	49.1	40.1, 58.2	2.5 (4.61)	1.22	1.29
Internet	282						
Irregular		147	52.6	42.9, 62.2	2.7 (4.92)	1.22	1.14
Regular/refugee/seeking asylum		130	47.4	37.8, 57.1	2.7 (4.92)	1.22	1.14

Abbreviations: CI, confidence interval; RDS, respondent-driven sampling; SE, standard error.

^a^ Irregular legal status refers to persons who were undocumented.

^b^ Regular legal status refers to persons with documentation, such as that related to temporary protected status or a visa.

**Figure 2:**
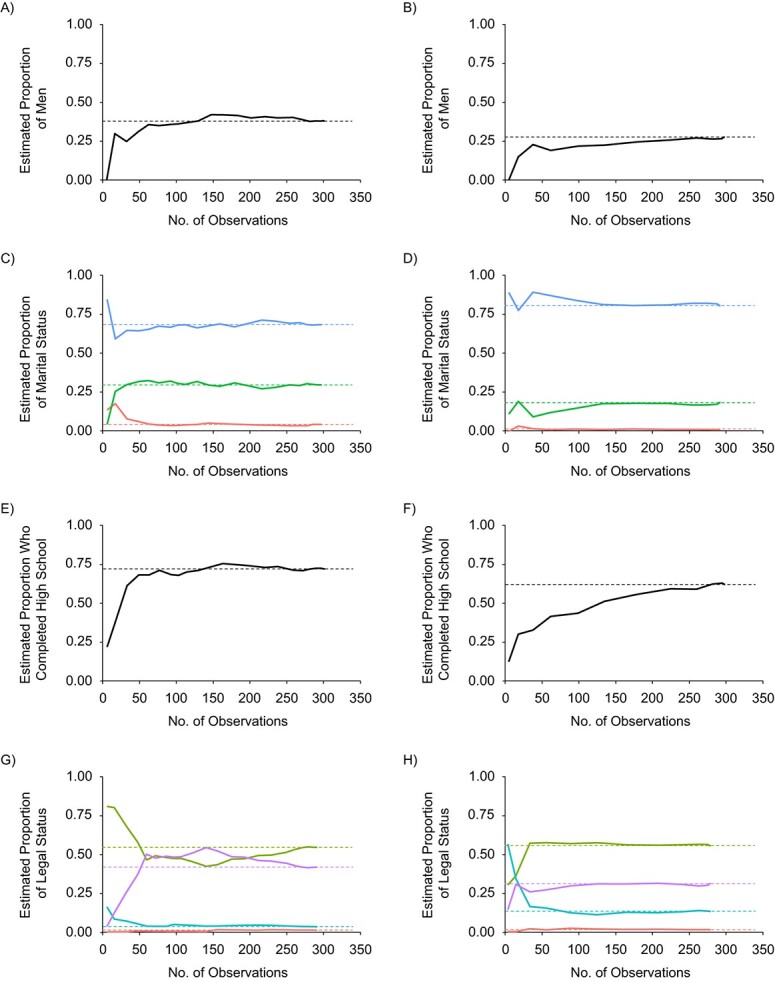
Convergence plots for selected demographic characteristics in a study of respondent-driven sampling (RDS) strategies among Venezuelan refugees and migrants, Bogotá, Colombia, 2020. The solid line represents estimates made as sampling occurs, whereas the dashed line shows the final estimate calculated once sampling has ended. The graph shows plots for male sex for phone RDS (A) and Internet RDS (B); marital status (single (blue), married/civil union/partner (green), or divorced/widowed/separated (red)) for phone RDS (C) and Internet RDS (D); high school completion for phone RDS (E) and Internet RDS (F); and legal status (irregular (i.e., undocumented; green), regular (i.e., documented; purple), refugee (blue), or asylum-seeker (red)) for phone RDS (G) and Internet RDS (H).

The results of convergence plots for select demographic characteristics are presented in Figure 2. In these plots, the vertical axis represents the participants as they enroll in the survey and the horizontal axis represents the final estimate. The solid line is the estimate as sampling occurs, whereas the dotted line is the final estimate once sampling has ended. Convergence is reached when the solid lines stabilize along the final estimate before sampling is completed. The solid line will always meet the dotted line by the end of sampling, but this does not mean that convergence has been reached.

For phone RDS, most of the convergence lines for the sociodemographic indicators rested on the dotted estimator lines. For Internet RDS, the convergence lines were near the estimate lines but did not actually reach convergence until sampling was finished. This indicates remaining dependence on the non–randomly selected seeds. Convergence appears to have been reached for both survey strategies for sex (Figures 2A and 2B). While convergence was not reached in the phone survey (Figure 2C), it was reached early on in the Internet survey for those currently living with a partner (Figure 2D). Convergence appears to have been reached for both survey strategies for education (Figures 2E and 2F) and legal status (Figures 2G and 2H).

**Figure 3:**
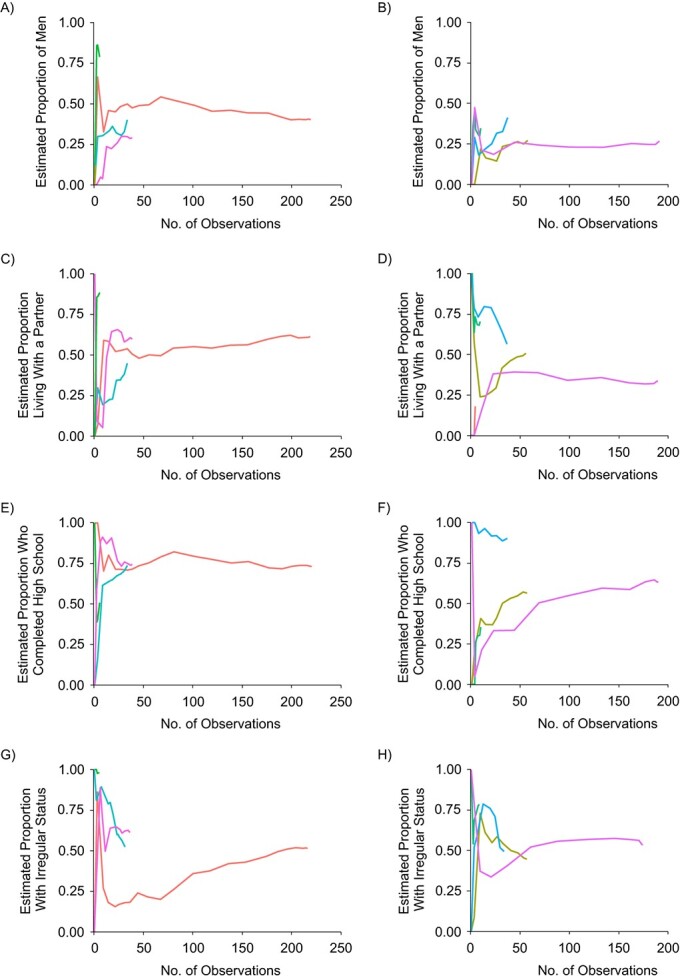
Bottleneck plots for selected demographic characteristics in a study of respondent-driven sampling (RDS) strategies among Venezuelan refugees and migrants, Bogotá, Colombia, 2020. Each colored line represents the convergence plot for an individual seed participant; convergence near a single population estimate indicates low homophily. The graph shows plots for male sex for phone RDS (A) and Internet RDS (B); marital status (married/civil union/partner) for phone RDS (C) and Internet RDS (D); high school completion for phone RDS (E) and Internet RDS (F); and irregular (undocumented) legal status for phone RDS (G) and Internet RDS (H).

We generated bottleneck plots (Figure 3) for 4 of the variables, but some seeds produced a much longer chain than others. Aside from impeding the ability to interpret bottleneck plots, it is neither unusual nor problematic in RDS samples for one seed to produce a much longer recruitment chain than other seeds. Ideally, to interpret whether there are no bottlenecks, all chains should move from their initial point toward the horizontal line indicating the final estimate for that variable. While it is unclear whether the shorter chains were moving toward the final estimates, the longest chains did not indicate any bottlenecks.

## DISCUSSION

**Figure 4:**
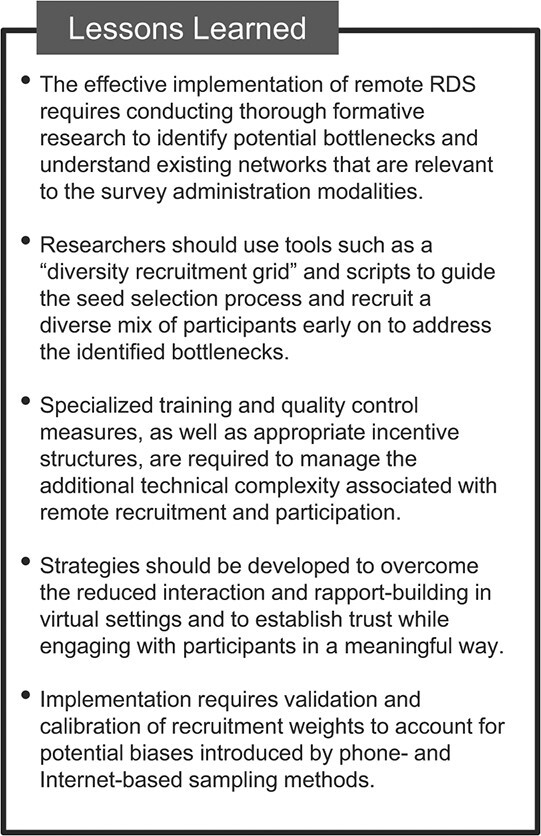
Lessons learned from a study of respondent-driven sampling (RDS) strategies among Venezuelan refugees and migrants, Bogotá and Norte de Santander, Colombia, 2020.

Remote RDS strategies are a feasible method with which to successfully recruit refugees and migrants in Colombia and to obtain quantitative estimates under certain conditions. The study was able to achieve its desired minimum sample size through phone-based recruitment at both survey sites and through the Internet in the city of Bogotá. The Internet survey approach in the remote border area of Norte de Santander did not succeed in reaching the desired minimum sample size. Formative research should be conducted to ensure that the following conditions are in place to successfully recruit and survey participants via the Internet (Figure 4). First, the population must be socially connected through Internet communication. Second, the population must have an electronic device with access to data or wireless fidelity (Wi-Fi; Wi-Fi Alliance, Austin, Texas) in order to receive, complete, and submit the Internet survey. Third, the population must have sufficient literacy to read the invitation and self-administer the survey, as well as have sufficient digital literacy to access and navigate the survey online. These conditions were not adequately met in the border region of Norte de Santander, where poorer access to electronic devices, less reliable Internet connections, lower digital literacy, and correspondingly lower digital social network connectivity in comparison with the capital city probably explains why the sample size was not reached for Internet RDS. Finally, as with phone RDS, the population must be willing to provide the phone numbers of their recruits, which may impede recruitment.

If the above requirements are not met and a remote method is needed, phone RDS provides an effective alternative. Phone interviews are similar to in-person interviews in that a trained interviewer administers the survey, although phone interviews lack the interpersonal features of face-to-face interaction. Phone RDS permits participation by eligible respondents who do not have Internet access or are illiterate, as long as they have a phone. Phone interviews, however, require answering a phone call and providing names and phone numbers of recruits, which may have made some respondents feel as though they were sharing personal information. An alternative is to use a passive method which requires participants to contact a hotline once they have been recruited by a peer (8). Whereas our active phone strategy took only 4 weeks to enroll 305 participants in Bogotá and 5 weeks to enroll 300 participants in Norte de Santander, the passive method used by Inghels et al. (8) took 9 months to recruit 518 participants and eventually stopped due to low enrollment.

There were additional challenges associated with remote RDS. Both phone and Internet strategies required careful tracking of the peer recruitment process. Phone RDS was easily monitored by the staff calling participants. Internet RDS required additional attention, as recruitment was done quickly through the Internet. For the 3 successful samples, recruitment resulted in long recruitment chains and convergence, with minimal homophily. Although homophily is fairly easy to calculate in RDS surveys, the level of impact any homophily has on the final estimates relies on other factors, including whether convergence has been met and whether bottlenecks are detected. Lacking convergence, having a bottleneck, and high homophily are likely evidence of bias, which can be explored qualitatively. In the Bogotá Internet survey, we found some population homophily for persons living with a partner; however, early convergence and no indication of a bottleneck for this variable probably mitigated bias in the estimates. While one recruitment chain had a much longer chain than others, the bottleneck plots and recruitment graphics did not indicate recruitment of any isolated subgroups. We attribute this to the rigorous formative research carried out to discover subgroups in the population’s social networks and the seed selection and direction instructions. The study did not enroll any ineligible persons owing to eligibility screening algorithms. During Internet RDS, we encountered instances of enrollment of more than 3 recruits due to unforeseen forwarding of invitations outside the recruitment parameters. In these instances, persons recruited outside the study’s recruitment channel were not be included and were removed from the database. We also encountered challenges in delivering incentives when the multiservice provider unexpectedly removed one of the main phone carriers from their services package, resulting in delays during the setup and verification process. Remote strategies require slight changes to measuring the network of the population, which is needed for the analysis of RDS data. Besides the measurement of networks through specific eligibility criteria, a temporal question was modified to ask, “How many of those who are in your social network have you been in contact with in person or via phone (calls/text) in the last 2 weeks?”.

In conclusion, remote RDS strategies for sampling hard-to-reach populations offer researchers valuable options for obtaining probability-based estimates. When commonly used in-person interviews and fixed-site RDS strategies are impossible, as was the case during the COVID-19 pandemic, remote online and phone options are feasible and are gaining interest among researchers. The remote RDS strategy (phone and Internet) saved participants the time, energy, and cost of going to a study location. It allowed for more physical anonymity than in-person surveys and reduced the risk of exposure to severe acute respiratory syndrome coronavirus 2 (SARS-CoV-2), the virus that causes COVID-19. Remote RDS should be explored further to estimate sampling error, overcome potential sampling bias due to lack of access to technology, and increase response rate and validity in face-to-face interviews. Innovative research approaches should be pursued to understand and quantify the risk and resiliency of hard-to-research populations such as refugees and migrants.

## Supplementary Material

Web_Material_kwad116
