# Supplementary material for: Innovative Strategies for Remotely Sampling Hard-to-Reach Populations: Assessing Phone Versus Internet Respondent-Driven Sampling Approaches Among Venezuelan Refugees and Migrants in Colombia
Source: Am J Epidemiol. 2023 May 16;192(10):1613–23. doi: 10.1093/aje/kwad116 (PMC10558185; doi:10.1093/aje/kwad116)
Supplement: Web_Material_kwad116 [file web_material_kwad116.zip › kwad116 Pham Web Material.pdf]

WEB MATERIAL

Innovative Strategies for Remotely Sampling Hard-to-Reach Populations: Assessing Phone Versus Internet Respondent-Driven Sampling Approaches Among Venezuelan Refugees and Migrants in Colombia

Phuong N. Pham, Lisa Johnston, Katrina Keegan, Carol Wei, and Patrick Vinck

Contents:

Web Table 1. Summary of phone versus Internet implementation with key lessons learned, Colombia, 2020

Web Table 2. Seed recruitment and diversity recruitment grid, Colombia, 2020

Web Table 3. Data security and management procedures, Colombia, 2020

Web Table 4. RDS assumptions and diagnostics conducted to assure assumptions are met

Web Table 5. Comparison of demographic characteristics of refugees and migrants by site, data collection methods, and recruiters and nonrecruiters in Bogotá, Colombia, 2020

Web Table 1. Summary of Phone versus Internet implementation with key lessons learned, Colombia, 2020

<table>
<tr><th>Study Step</th><th>Phone RDS</th><th>Internet RDS</th></tr>
<tr><td rowspan="2">STEP 1
Seeds: Seed selection and participation</td><td colspan="2">Local organizations facilitated contact with potential seeds. Potential seeds in each location were interviewed by phone using a diversity recruitment grid and phone script to map out potential recruits and determine whether potential seeds had diverse networks and could recruit those with targeted profiles. Those with the most diverse networks were selected as seeds and directed by interviewers to recruit pre-selected recruits to ensure diversity and inclusion of targeted profiles
Lessons learned and suggestions for future implementation: The initial pool of potential seeds identified was not large or diverse enough to initiate the surveys. We recommend establishing a robust database of at least three times the number of required seeds (one seed per 100 sample size) to ensure that additional seeds are available immediately when selected seeds do not respond or chains quickly die out. The use of a diversity grid and phone script specific to the sub-populations of interest are important to ensure that all subgroups are reached early in the sampling. In RDS the seeds introduce bias and are the only participants in the survey over which the research team has some control. Utilizing a diversity grid with seeds will encourage them to recruit across important subgroups, thereby overcoming bottlenecks and the exclusion of valuable characteristics.</td></tr>
<tr><td>Once selected, seeds were assigned an ID number and contacted and interviewed by phone by trained interviewers. Interviewers entered participants' responses in Kobo Toolbox. After the survey, interviewers read recruitment instructions and seeds identified and provided the contact information of three individuals identified in the diversity recruitment grid exercise. Peer-to-peer recruitment information, including ID numbers and contact information, was managed by research staff in an online database. Successively enrolled recruits were assigned ID numbers and linked to their recruiter.</td><td>Once selected, seeds were sent an invitation and unique survey link via WhatsApp and auto-completed the Internet survey via Kobo Toolbox. After the survey, seeds read recruitment instructions and entered WhatsApp numbers of three individuals identified in the diversity recruitment grid exercise. Peer-to-peer recruitment information was managed by research staff in an online database. Successively enrolled recruits were assigned ID numbers and linked to their recruiter.</td></tr>
</table>

STEP 2 Candidates: Initial contact/survey access	Using the contact information provided by a previous participant, a phone interviewer contacted a candidate. The interviewer read a script to introduce the study, informed the candidate that he/she was recruited by a peer to participate, and asked if it was a good time to talk or whether there was a better time to call back.<br>**Refusal:** If at any time the candidate said they did not want to participate, they were asked the reason why. The interviewer recorded their response in the survey. This helped the researchers discover if there are any particular reasons, such as the survey being too long, that could be remedied.<br>**Contacting persons:** The interviewer made no more than five attempts to make an initial contact with the candidate. If the candidate did not participate after five attempts, he/she was considered ineligible.<br>**Rescheduling calls:** If a candidate needed to reschedule a call, he/she agreed on a convenient time. Attempts were made to ensure the candidate was eligible before rescheduling. The interviewer made no more than five attempts to reschedule an interview.	Using the contact information provided by a previous participant, the study team sent an invitation and unique survey link via WhatsApp. The candidate clicked on the link to access the Kobo Toolbox Internet survey and read a script, which introduced the study and explained that he/she was recruited by a peer to participate.<br>**Refusal:** The candidate was able to stop the Internet survey at any time. From the onset, candidates and participants could access a website where they could contact the study team and leave feedback (including whether or not they completed the survey and why/why not). This helped the researchers discover if there were any particular reasons, such as the survey being too long, that could be remedied.<br>**Contacting persons:** The study team sent the candidate the invitation and survey link (first attempt), along with two follow-up reminder messages (second and third attempt). Each message was sent two days apart, thus spanning about a week. If the candidate did not participate after three attempts, they were considered ineligible.<br>***Lessons learned and suggestions for future implementation:*** *Ensure that there is a trained staff member who manages the WhatsApp account, communicating with participants and providing technical support. This was key not only in problem-solving but also establishing trust, as many people were wary of scams or pyramid schemes when they first received an invitation.*
STEP 3 Candidates: Eligibility screening	Interviewers used a script to introduce the eligibility section. Then, candidates answered a series of questions to determine eligibility.	Candidates read the introduction to the eligibility section and answered a series of questions to determine eligibility.
STEP 4 Candidates: Study information and consent	If the candidate was eligible, interviewers used a script to introduce the consent section. The interviewer read the study information and brief consent and directed participants to the website for the complete consent form if desired. The interviewer asked for the candidate's verbal consent, documented by the interviewer selecting yes or no in the survey. Once consent was granted, the candidate became a participant. If not, the candidate was thanked for his/her time and the call ended.	If the candidate was eligible, he/she was directed to a screen introducing the consent section. The candidate read the study information and brief consent form and could access the website for the complete consent form if desired. The candidate selected yes or no to indicate their consent. Once consent was granted, the candidate became a participant. If not, the candidate was thanked for his/her time and exited from the survey.

<table>
<tr><td>STEP 5
Participants:
Social network size</td><td colspan="2">The participant was introduced to the social network size section with the following message: “The first questions are about your network of friends and acquaintances. These are the most difficult questions that you will answer. Please take the time to think carefully about your answer, and answer as accurately as possible.” Participants then answered the following network size questions:
1. How many people do you know that are from Venezuela, who left Venezuela after 2014? We consider ‘knowing’ to mean that they know your name and you know theirs.
2. How many of these [# response from 1] persons are 18 years or older and have lived in [Bogotá or Norte de Santander], Colombia for at least one month?
3. How many of these [# response from 2] have you been in contact with in person or via phone (calls/text) in the last 2 weeks?
After the network size questions were answered, the participant completed the remaining part of the survey.
Lessons learned and suggestions for future implementation: Emphasizing the importance of the network size questions, cascading the question into more digestible parts, adding programmatic controls, and training phone interviewers (in the case of Phone RDS) are all useful strategies that ensure increased accuracy of the final network size number. Phone RDS may provide more accurate network sizes compared to Internet RDS since it requires being interviewed by a person who can probe for reliable responses. For Internet RDS, the questionnaire software for the social network size should be programmed to not allow zeros or numbers larger than that reported in the previous question (i.e., the response to question 2 should be the same or smaller than question 1). Employ these strategies to ensure that each participant’s network size is as accurate as possible, so that proper weights are applied during analysis.</td></tr>
<tr><td rowspan="2">STEP 6
Participants:
Recruitment</td><td colspan="2">Upon completion of the survey, participants were asked to recruit people they know who fulfilled eligibility criteria.</td></tr>
<tr><td>Using a script, interviewers introduced the recruitment section, briefly explained the recruitment process and incentive for successful recruitment and asked if the participant was willing to recruit up to three people.
If not, the interviewer continued to the incentive section.
If so, the interviewer explained the recruitment guidelines and asked participants to provide the name and phone numbers of their three recruits, which the interviewer entered into the survey form. These recruits were then contacted directly by phone interviewers.</td><td>Participants read the short explanation of the recruitment process and indicated whether or not he/she was willing to recruit up to three people.
If not, the participant continued to the incentive section.
If so, the participant read the recruitment guidelines and entered the phone numbers of their three recruits into the survey from. These recruits then received the invitation and survey link from the invitation manager.
Lessons learned and suggestions for future implementation: The team had not adequately prepared for the possibility of direct sharing of invitations between participants and their recruits, which created excess recruitment chains that were impossible to track. In hindsight, the research team should have invested in programming aimed at preventing duplicate survey submissions by different people under the same ID. It is critical that future iterations of Internet RDS programmed unique link per each invitation, thereby permitting only one submission.</td></tr>
</table>

STEP 7 Participants: Primary incentive	Participants received an explanation about incentives and provided the phone number and affiliated phone carrier to which they wished to have their incentive sent. Each participant who completed all study steps was provided an incentive (COP $12,000) for their time. This incentive was a mobile phone credit which was sent directly to the participant through Gana, a Colombian multi-service provider, within 30 hours of having successfully completed the survey.<br>***Lessons learned and suggestions for future implementation:*** *Options for the type, amount, and delivery method of incentives should be carefully considered for appropriateness and feasibility within individual contexts. Incentives must be provided timely to facilitate ongoing recruitment. In addition, it is important, no matter what virtual delivery method is chosen, to anticipate potential issues and develop contingency plans and protocols so incentives are provided in a timely manner. In this study, a mobile credit -delivered remotely- proved to be an effective alternative to cash incentive, especially in the face of the COVID-19 pandemic.*
STEP 8 Participants: Secondary incentive	Once all recruits had been contacted and either deemed participants or exited (steps 2-6), participants were sent a secondary incentive (COP $3,000) for each peer they recruited who successfully participated. The secondary incentive was a mobile phone credit sent directly to the participant through Gana, a Colombian multi-service provider, within 30 hours of their recruit's completing the survey.

Web Table 2. Seed recruitment and diversity recruitment grid, Colombia, 2020

**Seed** (Add row for each seed)	**Potential Recruit** (Add row for each seed's peer)	**Vulnerability Profile** Ensure representation of at least one of each targeted profile (in **bold**)						**Potential Bottlenecks** Ensure diversity among variables (e.g., not all male, not all in same neighborhood)					
		Family Situation/ Civil Status (select one)	Age (select one)	Disa-bility? (Y/N)	If Yes, Type: (select one)	Legal Status (select one)	UNHCR contact? (Y/N)	Sex (select one)	Education level (select one)	Employment status (select one)	Time in Colombia (select one)	Neigh-borhood (write-in)	Origin (write-in)
Seed A	Recruit 1	Single without children<br>Single with children<br>Married or in civil union<br>Divorced or widowed	18-19<br>20-29<br>30-39<br>40-49<br>50-59<br>60+	Yes<br>No	Vision<br>Hearing<br>Mobility<br>Commun-ication<br>Self-care<br>Cognition	Seeking asylum<br>Refugee<br>Irregular<br>Regular	Yes<br>No	Male<br>Female	None or pre-school<br>Primary (1st-5th)<br>Secondary (6th-11th) or technical<br>University or higher	Formal employment<br>Informal employment<br>Unemployed	<6 months<br>6 months-1 year<br>1-2 years<br>2-6 years		

Web Table 3. Data security and management procedures, Colombia, 2020

Activity	Phone RDS	Internet RDS
Data collection and management	• Survey responses reported over the phone were entered directly into Kobo Toolbox;<br>• Interviews were conducted in private spaces so responses to the survey were only heard by the interviewer;<br>• Participants were identified through unique ID numbers, rather than any personal information;<br>• PII (names and phone numbers) of participants (in Airtable) and their survey responses (in Kobo Toolbox) were kept separate to ensure privacy, confidentiality and data security.<br>• Only the RDS study team had access to survey responses. The phone interviewers and supervisors had access to select information in the online database during study implementation and access was removed after study completion;<br>• Upon the conclusion of the study, all names and phone numbers were removed from the online database and the website was taken down	• Participants were identified through unique ID numbers, rather than any personal information;<br>• PII (WhatsApp numbers) of participants (in the online database) and their survey responses (in Kobo Toolbox) were kept separate to ensure privacy, confidentiality and data security.<br>• Only the RDS study team had access to survey responses and the online database.<br>• Upon the conclusion of the study, all phone numbers were removed from the online database, the WhatsApp account (and all contacts) were deleted, and the website was taken down
Recruitment and contacting seeds and recruits	• Recruits were called and asked if they wanted to participate at that time or to reschedule;<br>• Only five attempts were made to make initial contact with a recruit or to reschedule an interview	• Researchers created and used a WhatsApp business account to send invitations and reminder messages and provide technical support to participants as needed;<br>• A designated staff member managed the account and communication with participants (identified by their ID) exclusively through the designated WhatsApp account;<br>• Participants received unique survey links associated with their ID (no PII)
Incentives	• Incentives were released through a secure platform (Gana)	
Report development	• Reports and publications were developed by researchers;<br>• Data generated by the survey for analysis did include any PII (names or phone numbers)	• Reports and publications were developed by researchers;<br>• Data generated by the survey for analysis did not include any PII (WhatsApp numbers).

Web Table 4. RDS assumptions and diagnostics conducted to assure assumptions are met

Assumption	Assumption Definition	Diagnostic	Diagnostic Definition and Importance	Problems When Assumptions Are Not Met
Seed dependence	RDS requires that the final estimates are no longer dependent on the non-randomly selected seeds.	Convergence plot: The plot shows that the number of subjects enrolling in the survey will converge along the final estimate in advance of the final sample being reached.	Like other peer-to-peer recruitment methods, the population sampled must be socially networked. The RDS sampling approach begins with a set of non-randomly selected initial individuals who meet the eligibility criteria (referred to as seeds). These initial recruits enroll in the survey and then are provided a small number (usually three) of coupons to use in recruiting their peers. Unlike most peer-to-peer sampling methods, RDS extends recruitment over as many waves as possible (ideally more than six), thereby eliminating bias from the original non-randomly selected seed. Once the sample size is reached, this 'non-independence,' known as convergence, can be measured (13,21).	If convergence is not met, it is an indication that recruitment has not become random and that the final estimates may be biased.
Sample is one complete network component	RDS requires that the sample be connected as one network component. This means that subgroups within a population should be linked together.	- Recruitment graphics: Recruitment graphics of a particular trait (i.e., age groups) in the final sample should show that trait as being dispersed throughout the recruitment chains.<br>- Bottleneck plots: Plots should demonstrate that the recruitment chains are moving in the direction of the final estimate of a trait.	A potential bias in RDS is when a population is composed of numerous subgroups which are not connected, thereby creating 'bottlenecks.' Bottlenecks are problematic if each distinct subgroup has significantly different estimates on important variables. Bottlenecks can be assessed through plots of recruitment on variables of interested and through recruitment graphics (13,21).	If the sample shows a bottleneck with two subgroups showing up in distinct chains, then the sample is likely not one sample but two samples. Bottlenecks may be an indication that the final estimates are biased.
Statistical dependence Among subgroups	Individuals should not show strong ties to those similar or dissimilar to themselves.	Recruitment and population homophily: A value of 1 is an indication of no homophily or heterophily. Higher values and lower values may indicate high homophily or heterophily.	In RDS, data are collected on who recruited whom in order to measure random recruitment, measured as homophily and heterophily (7). High homophily indicates recruitment preferences for those who are similar and high heterophily indicates preferences for those who are dissimilar. Recruitment homophily shows preferences in the recruitment process and population homophily shows preferences in the networked population.	High homophily (when groups purposively recruit their same type: females only recruit females) or heterophily (females only recruit males) is an indication of possible bias in either the sample recruitment or population recruitment. High homophily/heterophily may result in biased estimates.
The population is socially networked	For peer-to-peer recruitment to work, the population must be socially networked.	A final sample made up of long recruitment chains.	A sample ending up with short and fat recruitment chains are an indication that the population is not socially networked.	Final estimates will not be accurate.

Web Table 5. Comparison of demographic characteristics of refugees and migrants by site, data collection methods, and recruiters and nonrecruiters in Bogotá, Colombia, 2020

Variable	Bogotá-Overall					Bogotá-Phone					Bogotá-Internet				
	Phone *n* = 305		Internet *n* = 302		*P* Value	Recruiter *n* = 228		Nonrecruiter *n* = 77		*P* Value	Recruiter *n* = 235		Nonrecruiter *n* = 67		*P* Value
	No.	% (95% CI)	No.	% (95% CI)		No.	% (95% CI)	No.	% (95% CI)		No.	% (95% CI)	No.	% (95% CI)	
**Sex**															
Male	127	41.6 (32.7, 51.1)	79	25.5 (18.8, 33.6)	0.008	97	47.4 (36.7, 58.3)	30	31.0 (17.3, 49.0)	0.117	53	17.7 (12.1, 25.1)	26	44.8 (28.4, 62.3)	0.002
Female	178	58.4 (48.9, 67.3)	222	74.5 (66.4, 81.2)		131	52.6 (41.7, 63.3)	47	69.0 (51.0, 82.7)		182	82.3 (74.9, 87.9)	40	55.2 (37.7, 71.6)	
**Marital Status**															
Living with a Partner (yes)	181	60.4 (51.2, 68.9)	135	42.2 (34.0, 50.9)	0.005	140	61.8 (50.8, 71.6)	41	57.9 (40.9, 73.1)	0.698	111	44.7 (35.1, 54.8)	24	36.2 (21.4, 54.0)	0.399
**Civil Status**															
Divorced, Separated, or Widowed	12	3.2 (1.0, 10.0)	6	2.6 (0.6, 11.5)	0.074	10	1.7 (0.9, 3.3)	2	6.0 (1.0, 27.8)	0.313	4	0.6 (0.2, 1.7)	2	7.4 (1.2, 35.1)	0.049
Married or Civil Union	92	33.8 (25.6, 43.1)	60	18.9 (13.1, 26.6)		71	31.7 (22.8, 42.1)	21	37.6 (22.5, 55.6)		49	17.0 (11.7, 25.1)	11	22.5 (10.3, 42.4)	
Single	196	62.9 (53.5, 71.5)	230	78.5 (70.1, 85.0)		143	66.6 (56.2, 75.6)	53	56.4 (38.9, 72.4)		178	82.0 (74.3, 87.8)	52	70.1 (49.5, 84.8)	
**Education**															
Secondary school or less	65	18.7 (13.1, 26.0)	90	28.5 (21.3, 37.1)	0.059	50	19.2 (12.6, 28.1)	15	17.8 (8.9, 32.4)	0.843	72	27.7 (19.7, 37.4)	18	30.5 (16.6, 49.2)	0.766
High school or more	240	81.3 (74.0, 86.9)	212	71.5 (62.9, 78.7)		178	80.8 (71.9, 87.4)	62	82.2 (67.6, 91.1)		163	72.3 (62.6, 80.3)	49	69.5 (50.8, 83.4)	
**Legal Status**															
Seeking asylum	4	2.8 (0.7, 10.8)	6	1.3 (0.4, 4.1)	0.175	4	4.2 (1.0, 15.9)	0	0	0.198	6	1.9 (0.6, 5.7)	0	0	0.008
Refugee	15	4.6 (1.9, 11.0)	24	10.6 (6.3, 17.4)		13	6.7 (2.6, 16.2)	2	0.7 (0.2, 3.0)		19	10.1 (5.5, 18.0)	5	11.9 (4.4, 28.6)	
Irregular (undocumented)	131	46.2 (37.1, 55.5)	149	51.5 (42.5, 60.4)		96	41.7 (31.6, 52.6)	35	54.6 (37.2, 70.9)		124	59.9 (49.4, 69.5)	25	30.3 (17.1, 47.7)	
Regular (documented)	145	46.4 (37.3, 55.8)	103	36.5 (28.1, 45.8)		108	47.3 (36.7, 58.2)	37	44.7 (28.4, 62.2)		72	28.1 (19.7, 38.4)	31	57.8 (40.0, 73.9)	
